# Evaluation of accompanying allergic disease in children with proven drug allergies

**DOI:** 10.55730/1300-0144.5793

**Published:** 2023-11-18

**Authors:** Şule BÜYÜK YAYTOKGİL, Kezban İPEK DEMİR, Özge YILMAZ TOPAL, Azize Pınar METBULUT, İlknur KÜLHAŞ ÇELİK, Müge TOYRAN, Ersoy CİVELEK, Emine DİBEK MISIRLIOĞLU

**Affiliations:** Department of Pediatric Allergy and Immunology, University of Health Sciences, Ankara City Hospital, Ankara, Turkiye

**Keywords:** Allergic disease, allergic rhinitis, asthma, atopy, atopic dermatitis, drug hypersensitivity reactions

## Abstract

**Background/aim:**

Data on the prevalence of allergic diseases in children with proven drug allergies are limited. We aim to evaluate the frequency of allergic comorbidity in children with proven common drug allergies.

**Materials and methods:**

Children with drug hypersensitivity confirmed by diagnostic allergy tests at our center between January 2010 and December 2020 were screened retrospectively. Patients with the most common drug allergies (due to antibiotics, nonsteroidal antiinflammatory drugs [NSAIDs], and antiepileptic drugs) were selected for analysis. Age, sex, the culprit drug, initial reaction characteristics, diagnostic test results, and the study physician who diagnosed concomitant allergic diseases were noted.

**Results:**

A total of 168 patients (boys, 51.2%) with a median age of 12 years (IQR = 8–16.3) were included in the study. The culprit drug was an antibiotic in 63% (n = 106), NSAID in 25% (n = 42) and anticonvulsant in 11.9 % (n = 20) of the patients. Drug hypersensitivity reactions were immediate in 74.4 % (n = 125) and delayed in 25.6 % (n = 43) of the patients. Seventy-five patients (44.6 %) had at least one allergic disease, most commonly rhinitis (27.3 %, n = 46) or asthma (25 %, n = 42). Fifty-five patients underwent skin prick tests with aeroallergens, producing a positive result in 60% (n = 31). The prevalence of allergic disease was not differing according to the culprit drug. The frequency of developing at least one concomitant allergic disease was 47.2% (n = 50/106) for antibiotic hypersensitivity, 52.4% (n = 22/42) for NSAID hypersensitivity, and 15% (n = 3/20) for anticonvulsant hypersensitivity (p < 0.00).

Immediate drug hypersensitivity reactions were more frequent in children who had allergic diseases (80 % vs. 64.5 %; p = 0.027).

**Conclusion:**

Nearly half (44.6%) of the children with proven drug hypersensitivity had concomitant allergic diseases and immediate reactions were more common in this group. Children evaluated for drug hypersensitivity should be assessed for other allergic diseases.

## 1. Introduction

Drug hypersensitivity reactions (DHRs) are seen in 10% of children [1], although few (4.4%–6.9%) can be proven [1–4]. Beta-lactam antibiotics are the most frequent culprit drugs in children with drug allergies, followed by nonsteroidal antiinflammatory drugs (NSAIDs) and nonbetalactam antibiotics [2–4]. Antiepileptic drugs (AEDs) are another frequent cause of DHRs, especially severe reactions [5]. DHRs are diagnosed and proven (i.e. the culprit drug is identified) using clinical history, skin tests, and drug provocation test (DPT)[1,6,7].

The presence of allergic disease, such as food allergy (FA), atopic dermatitis (AD), allergic rhinitis (AR), or asthma, increases the risk of developing other allergic diseases [8]. This relationship has been described as the “atopic march”. Although drug allergies were not included in this definition (perhaps because of the mechanisms involved), recent studies have indicated that allergic disease is one of the risk factors for and may coexist with drug allergies [9–12]. However, in the literature (English), there are few studies on the frequency of allergic diseases or aeroallergen hypersensitivity in children with proven drug allergies and the data available come from studies involving small patient groups. Therefore, our aim in this study was to evaluate the frequency of allergic diseases (FA, AD, AR, and asthma) in children with proven drug allergies.

## 2. Materials and methods

### 2.1. Study population

This study included patients younger than 18 years of age with NSAID, antibiotic, and anticonvulsant allergies confirmed by allergological work-up in the pediatric allergy outpatient clinic of our hospital over a 10-year period (January 2010–December 2020). The study was approved by the ethics committee of Ankara City Children’s Hospital (decision number: E2-21-148), and informed consent was obtained from parents.

### 2.2. Study procedures

We retrospectively screened the patients’ medical records and used a standard form to record the patients’ demographic, atopic, and clinical characteristics. The presence of allergic comorbidities (FA, AD, AR, and asthma) diagnosed by the study physicians was noted. The patients’ parents were contacted by phone to obtain missing data. Patients whose history of allergic disease was unclear or whose drug allergy was not confirmed with allergological tests were excluded from the study.

### 2.3. Confirmation and classification of drug hypersensitivity reactions

Patients who presented with suspected drug allergy were evaluated according to history and clinical presentation and underwent skin tests and/or DPT with the culprit drug. Reactions occurring within 24 h for NSAIDs and within 1-h for other drugs were classified as immediate reactions; those that occurred later were classified as non immediate (delayed) reactions [6, 13]. Anaphylaxis was defined according to the criteria suggested in the European Academy of Allergy and Clinical Immunology Task Force position paper on the management of anaphylaxis in childhood [14]. For patients whose history was consistent with an immediate reaction, skin tests (skin prick test and intra-dermal test) and DPT with the suspected drug were performed. For those whose history suggested a delayed reaction, skin tests (patch test and intra-dermal test with late reading) and/or DPT with the suspected drug were performed. The tests were done after one month for other reaction types. Antihistamine medications and other drugs that could affect skin tests or DPT were discontinued one week before testing. Written informed consent was obtained from the caregivers before all tests.

### 2.4. Definition and diagnosis of allergic diseases

Information related to concomitant allergic diseases diagnosed by the study physicians (all pediatric allergy immunology specialists) was evaluated from the patients’ records.

### 2.5. Food allergy

FA diagnosis was based on clinical history and laboratory tests (specific IgE [sIgE] test and skin prick test) and/or food challenge tests, in accordance with the relevant guidelines [15]. Skin prick tests were performed to detect IgE-mediated sensitization using commercially available solutions (ALK-Albelló, Madrid, Spain) or fresh forms of food (prick-to-prick). Food-specific IgE antibodies were analyzed in inpatient serum samples using the immunoCAP (Phadia; AB, Uppsala, Sweden)/IMMULITE (Siemens Healthcare Diagnostics, Tarrytown, NY, USA) test. An oral food challenge was also performed in patients with a suspected single FA according to guideline recommendations based on the patient’s age and reaction [16].

### 2.6. Asthma

Asthma was diagnosed according to the Global Initiative for Asthma (GINA) criteria [17]. In patients over five years of age, asthma was diagnosed with clinical history, physical examination, the reversibility in respiratory function test, and response to medication. In patients younger than five years of age, asthma was diagnosed with clinical history, physical examination, and response to medication.

### 2.7. Rhinitis/allergic rhinitis

AR was diagnosed based on typical symptoms (nasal blockage, discharge, itching/sneezing, and eye symptoms) and/or allergy confirmation with an sIgE and/or skin prick test [18]. Skin tests were performed with aeroallergens such as *Dermatophagoides-farinae, Dermatophagoides-pteronyssinus*, *Alternaria, Aspergillus, Cladosporium*, a cockroach, a cat, a dog, *Artemisia, Parietaria, Secale*, treemix, Oleaceae, and grasses. In addition, an sIgE panel for grasses and mites (*D. farina* and *D. pteronyssinus*) was used. We also included patients who had rhinitis symptoms (rhinorrhea, sneezing, itchy nose, and nasal congestion) that resolved with nasal steroids but could not be tested because of COVID-19 pandemic restrictions.

### 2.8. Atopic dermatitis

AD was diagnosed based on clinical history and objective findings (morphologic lesion features) in accordance with the diagnostic criteria of the American Academy of Dermatology [19].

### 2.9. Statistical analysis

The data were analyzed using SPSS version 22.0 (IBM Corp, Armonk, NY). Categorical values that were not normally distributed were presented as medians and inter quartile ranges (IQRs; 25th–75th percentiles). A p-value of <0.05 was considered significant.

There was no control group in our study, so we compared our results with those of previous studies (national and world data) [20–22]. A power analysis of the study was performed retrospectively with Open Epi v3 [23] (an open-source calculator), and our study sample size resulted in greater than 99% power.

## 3. Results

We evaluated 168 patients with confirmed antibiotic, NSAID, and AED allergies for concurrent IgE-mediated allergic diseases. The patients’ median age was 12 years (IQR: 8–16.3), and 51.2 % (n = 86) were boys. The baseline characteristics of the patients are summarized in Table 1.

The median age of the patients at theinitial drug reaction was seven years (IQR: 4–11.3). The median time from the initial reaction to diagnosis was 3.5 months (IQR: 2–10.2). The culprit drug was an antibiotic in 106 patients (63.1%), NSAIDs in 42 patients (25%), and AED in 20 patients (11.9%). Reactions were immediate in 125 patients (anaphylaxis in 40 patients) and delayed in 43 patients. Thirteen patients had severe cutaneous adverse reactions (Stevens-Johnson syndrome in six, DRESS in five, and acute generalized exanthematous pustulosis in two patients). The characteristics of the patients and their DHRs are summarized in Table 1.

Seventy-five (44.6 %) of the 168 patients had an allergic disease (shown in Figure). Forty-two patients (25%) had asthma, 46 (27.3%) had rhinitis (proven AR in 27 patients), 13 (7.7%) had AD, and 10 (5.9%) had FA. Sixty (48%) of the 125 patients who had immediate reactions had allergic diseases. Based on the reaction types, we observed that concomitant allergic diseases were more common in patients with immediate DHRs compared to those with delayed DHRs (48% vs. 31%; p = 0.027). Also, 14 patients had chronic urticaria.

Fifty-eight of the 168 patients were tested for sensitivity to aero allergens. Skin tests with aeroallergens were performed in 55 patients, producing a positive result in 33 of them. Pollen allergy was the most common (n = 24). Six patients were tested with the sIgE panel, and three of them had a positive result. The distribution of allergic diseases according to the culprit drug group is shown in Table 2.

When we compared patients with and without allergic diseases, there was no difference in terms of median current age, age at the initial reaction, sex, or family history of drug allergies (p > 0.05). However, patients with allergic diseases had higher rates of immediate DHRs (80% vs. 64.5%; p = 0.027) and a family history of atopy (36% vs. 12.9 %; p < 0.001) than patients without concomitant allergic diseases (Table 3).

## 4. Discussion/Conclusion

In this study, we evaluated the prevalence of concomitant pediatric allergic diseases, diagnosed by allergy immunology specialists, in 168 children with proven drug allergies. We determined that approximately half of the patients had allergic diseases in addition to drug allergies.

Allergic diseases are estimated to affect approximately 25% of the population in developed countries [21]. Similarly, the cumulative prevalence of allergic diseases in Turkish children was previously reported as 23.4% [22, 23]. The evidence indicates that an allergic disease, such as FA, AD, AR, and asthma, increases the risk of developing other allergic diseases and drug allergies. In a previous study, patients with beta-lactam allergy were reported to have a higher frequency of atopy with inhalant allergens (especially mites) when compared to the control group [11]. In a study by Duqueetal [24] investigating the risk factors for drug allergy in 26 Chinese pediatric patients with confirmed drug allergies, at least one concomitant allergic disease was present in 69.2 % of the children (AD in 30.8 %, AR in 42.3%, and asthma in 26.9%), compared to a prevalence rate of 51% in children in a control group without drug allergies. Indarat et al. [25] reported that 32.3% of 34 pediatric patients with DPT-confirmed drug allergies had a concomitant allergic disease (AR in 29.4%, asthma in 20.5%, AD in 8.8%, Chronic Urticaria in 8.8%, and FA in 2.9%).

However, the small groups evaluated in these studies make it difficult to evaluate and generalize the results. In our study, which included a fairly large sample of patients with proven drug allergies compared to previous studies, we determined that nearly half of the 168 pediatric patients had at least one concomitant allergic disease. Of these patients, 27.3 % (n = 46) had rhinitis, 25 % (n = 42) had asthma, 7.7 % (n = 13) had AD, and 5.9% (n = 10) had FA. Although we did not include a control group for comparison, the frequency of allergic diseases in children with drug allergies is higher than frequencies previously reported for our country and worldwide (cumulative prevalence of allergic diseases: 23.4% in Turkey [22,23] and 20%–25% globally [21,26]; cumulative prevalence of asthma: 6.9%–12.6%, AR: 11.7%–13.6%, allergic conjunctivitis: 11.7%, and atopic eczema: 2.6 %–8.3 % [22], according to studies from Türkiye). McFadden et al. [27] previously suggested that the hapten-atopy hypothesis may be related to this context. The authors posited that the cause of the dramatically increasing frequency of atopic diseases may be an increase in exposure to dietary chemical and drug haptens via processed foods, formula milk, food preservatives, and oral antibiotics and drugs in the environment. McFadden et al. [28] added that exposure to some types of haptens may change the immunological environment and affect TH2 immune responses.

Some studies exploring the relationship between drug allergies and other allergic diseases have suggested that drug allergies may be induced by IgE-mediated allergic diseases [10, 11, 29, 30]. In their study conducted in Spain, Cornejo-Garcia et al. reported that atopy, high total IgE levels, the presence of sIgE against mites, and IL4RA polymorphisms were predictors of immediate-type beta-lactam allergy [11]. Similarly, Choı Is et al. [10] reported a significant correlation between a positive drug allergy history and AR, night cough, FA, skin allergy, and sensitization with house dust mites. The authors noted a high frequency of IgE-mediated drug reactions. Similarly, based on the reaction types in our study, we observed that concomitant allergic diseases were more common in patients with immediate DHRs compared to those with delayed DHRs (48% vs. 31%). Although these data support the hypothesis that IgE-mediated allergic diseases can induce drug allergies, further studies are needed to clarify the causative relationship between drug allergy and allergic diseases.

In our study, we also compared the patients according to culprit drug groups. In our review of the literature (English), we found few studies evaluating the frequency of concomitant allergic diseases in patients with drug hypersensitivity according to drug group [12]. Kidon et al. [12] evaluated children with NSAID allergies in comparison to a control group of children with antibiotic allergies and found that asthma was more common among children with NSAID hypersensitivity than in those with antibiotic hypersensitivity. In our study, a comparison of patients in the three-drug groups (NSAID, antibiotic, and AED) revealed that the frequency of at least one allergic disease was 52.4 % in children with NSAID hypersensitivity (n = 22/42), 47.2% in those with antibiotic hypersensitivity (n = 50/106), and only 15% (n = 3/20) in those with antiepileptic hypersensitivity. Although the frequency of asthma and other concomitant allergic diseases was higher in the NSAID group compared to the antibiotic group, the difference did not reach statistical significance, contrary to the Kidon et al.’s [12] findings. More studies are needed regarding the coexistence of NSAID and antibiotic allergies and other allergic diseases.

NSAIDs are among the most studied drug groups in terms of the frequency of Concomitant allergic diseases [12, 31]. Sanchez-Borges et al [31] detected the atopic disease in 82% of 50 patients aged 8–63 years with confirmed NSAID hypersensitivity, compared to only 14.5 % of 48 adults in the control group. The authors also reported that aeroallergen sensitivity, detected by a skin prick test, was more common in the NSAID group compared to the control group (86.6% vs. 29.1%; p = 0.0001). Kidon et al. [12] evaluated 24 pediatric patients with DPT-proven NSAID allergies and reported that 83% had a concomitant allergic disease (AR in 58%, asthma in 46%, and AD in 29%) and 88% was sensitive to at least one aeroallergen (detected by a skin prick test). In our study, we found that 52.4% of patients with NSAID hypersensitivity had at least one allergic disease (rhinitis in 35.7%, asthma in 31%, FA in 4.8 %, and AD in 4.8 %). Furthermore, of the 18 patients in the NSAID group tested for aeroallergen sensitivity (17 by askin prick test and 1 by asIgE test), aeroallergen atopy was detected in 13.

The frequency of concomitant allergic disease and/or atopy in patients with beta-lactam antibiotic hypersensitivity has also been investigated in a limited number of studies [9, 32]. Apter et al. [9] reported a history of allergic disease in 17 (74%) of 23 patients (asthma in 57%, AR in 70%, and eczema in 17%). However, drug allergies were not confirmed by diagnostic tests in their study. Faitelson et al. [32] reported a history of atopy in 22% (n = 29) of 133 children referred to their clinic for suspected amoxicillin allergy. However, the allergy was confirmed by DPT in only 10 of the 133 patients, and this group had a higher frequency of asthma and FA. Asthma emerged as an important risk factor for confirmed amoxicillin allergy in the authors’ multivariate analysis. Our study included 106 patients with confirmed antibiotic allergy (beta-lactam in 104 cases), approximately half of whom (47.2%) had at least one concomitant allergic disease (rhinitis in 29.2%, asthma in 24.5%, AD in 10.4%, and FA in 7.5%). Aeroallergen sensitivity was detected in 21 of the 38 patients with antibiotic allergies (36 underwent a skin prick test and 2 underwent sIgE test).

This study has certain limitations. First, there was no control group for a comparison of allergic disease frequencies. However, the rates in our study group are well above the prevalence of allergic disease in the general population of Türkiye reported in previous studies. A second limitation is that some of the patients could not undergo skin prick tests for aeroallergen sensitivity due to the COVID-19 pandemic and the retrospective nature of the study.

In contrast, the strengths of our study are that allergic diseases were diagnosed by pediatric allergy immunology specialists who examined the patients in the allergy clinic, not according to patient or family reports, and the data reflect a single-center experience (i.e. all patients in the sample were followed using the same procedure). In addition, despite the limitations, our results make an important contribution to the literature because there are limited data on the prevalence of concomitant allergic diseases in children with drug allergies, and the study included a relatively large number of pediatric patients with confirmed drug allergies compared to similar studies in the literature.

In conclusion, our results support existing evidence that allergic diseases may be more common in children with drug allergies, especially among patients with immediate DHRs, than in the general population. This should be considered during history-taking and follow-up of pediatric patients with drug allergies, especially to antibiotics and NSAIDs, and detailed evaluation for other allergic diseases should be performed for patients with a suggestive history or signs.

## Figures and Tables

**Figure:**
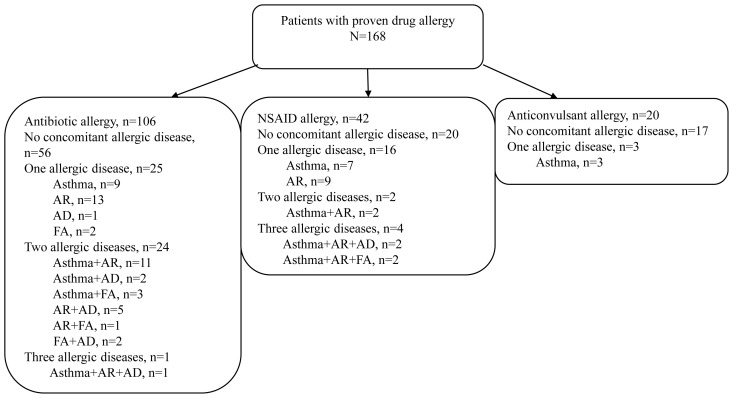
Concomitant allergic diseases in children with proven drug allergies according to types of drug.

**Table 1:** Baseline characteristics of the patients and reactions (n: 168).

Age (months), median (IQR)	12 (8–16.3)
Male, n (%)	86 (51.2)
Family history of allergic disease, n (%)	39 (23.2)
Previous drug allergy, n (%)	17 (10.1)
Family drug allergy, n (%)	18 (10.7)
**Reaction characteristics**	
Culprit drug, n	168
Antibiotic, n (%)	106 (63.1)
Penicillin, n	2
Aminopenicillin, n	67
Cephalosporin, n	35
Other (vancomycin, trimethoprim sulfametoxazol), n	2
NSAID, n (%)	42 (25)
Ibuprofen, n	27
Paracetamol, n	11
Other (aspirin, metamizole sodium), n	4
Anticonvulsant, n (%)	20 (11.9)
Carbamazepine, n	12
Other (valproate, phenobarbital, lamotrigine, midazolam), n	8
Age at reaction (years), median (IQR)	7 (4–11.3)
Time from reaction to diagnosis (months), median (IQR)	1.5 (2–10.2)
Diagnostic test for proven of DHR, n (%)	
Oral provocation test, n (%)	105 (62.5)
Skin prick test, n (%)	9 (5.4)
Intradermal test, n (%)	28 (16.7)
Patch test, n (%)	26(15.5)
Drug anaphylaxis, n (%)	40 (23.8)
Reaction type, n (%)	
Immediate, n (%)	125 (74.4)
Delayed, n (%)	43(25.6)
SCAR, n (%)	13(7.7)
DRESS, n (%)	5
SJS, n (%)	6
AGEP, n (%)	2

IQR: Interquartile range, NSAID: Nonsteroidal antiinflammatory drug,

DHR: Drug hypersensitivity reaction

**Table 2:** Distribution of allergic diseases according to type of culprit drug.

	Asthma	Allergic rhinitis	Food allergy	Atopic dermatitis	Allergic disease
Antibiotic n = 106, n(%)	26 (24.5)	31 (29.2)	8 (7.5)	11(10.4)	50 (47.2)
NSAID n = 42, n (%)	13 (31)	15 (35.7)	2 (4.8)	2 (4.8)	22 (52.4)
Anticonvulsant n = 20, n (%)	3 (15)	0	0	0	3 (15)

NSAID: Nonsteroidal antiinflammatory drug

**Table 3:** Comparison of patients with and without allergic disease.

	Allergic disease (n = 75)	No allergic disease (n = 93)	P
Age at the reaction (months), median (IQR)	7(4–11)	7 (3–12)	0.972
Immediate, n(%)	60 (80)	60 (64.5)	0.027
Anaphylaxis, n(%)	19 ( 25.3)	21 (22.5)	0.677
SCAR, n(%)	1 (0.01)	12 (12.9)	0.007
Antibiotic, n (%)	50 (66.6)	56 (60.2)	0.389
NSAID, n (%)	22 (29.3)	20 (21.5)	0.244
Anticonvulsant, n (%)	3 (4)	17 (18.2)	0.005
Family drug allergy, n (%)	11 (14.6 )	7 (7.5)	0.137
Family allergic diseases, n (%)	27 ( 36)	12 ( 12.9)	0.000
Multiple drug allergy, n (%)	9 ( 12)	8 (8.6)	0.468
Recent age (months), median (IQR)	12 (9–16)	12(7–17)	0.455
Gender (male), n (%)	37(49.3)	49 (52.6)	0.665

IQR: Interquartile range, DHR: Drug hypersensitivity reaction,
